# Influence of Host’s Plant Diet on Gut Microbial Communities and Metabolic Potential in *Spodoptera frugiperda*

**DOI:** 10.3390/insects16101042

**Published:** 2025-10-10

**Authors:** Wan-Ying Dong, Muhammad Hafeez, Sheng-Yuan Zhao, Jin-Ming Zhang, Muhammad Imran, Farman Ullah, Xiao-Wei Li, Yao-Bin Lu

**Affiliations:** 1 State Key Laboratory for Quality and Safety of Agro-Products, Key Laboratory of Biotechnology in Plant Protection of MOA of China and Zhejiang Province, Institute of Plant Protection and Microbiology, Zhejiang Academy of Agricultural Sciences, Hangzhou 310021, China; dongwy@zaas.ac.cn (W.-Y.D.); zhanginsect@163.com (J.-M.Z.); farmanullah787@gmail.com (F.U.); 2 Department of Agriculture, Veterinary, and Rangeland Science, University of Nevada Reno, Reno, NV 89557, USA; mhafeez@unr.edu; 3 Institute of Bio-Interaction, Xianghu Laboratory, Hangzhou 311231, China; zhaosy90@126.com; 4 Department of Chemistry, Faculty of Science, King Khalid University, P.O. Box 9004, Abha 61413, Saudi Arabia; imranchemist@gmail.com; 5 Xinjiang Key Laboratory of Agricultural Biosafety, Urumqi 830091, China

**Keywords:** gut microbiota, *Spodoptera frugiperda*, host plant diet, microbial diversity, metabolic profiling, plant–insect–microbe interactions

## Abstract

The gut microbiota of insects, shaped by extensive coevolution, plays a crucial role in host adaptability and ecological expansion. The fall armyworm (*Spodoptera frugiperda* J.E. Smith), a highly polyphagous and migratory invasive pest, infests more than 350 plant species worldwide, resulting in substantial crop losses and increased reliance on pesticide applications. In this study, we employed 16S rRNA high-throughput sequencing to investigate diet-induced alternations in the gut microbial communities of larvae fed corn, rice, or an artificial diet. The results showed that diet significantly influenced microbial diversity, with rice-fed larvae exhibiting the highest richness. Firmicutes, Proteobacteria, and Cyanobacteria were the predominant bacterial phyla. Genus-level analysis revealed notable diet-dependent shifts, with *Enterobacter* and other genera (e.g., *Ochrobactrum* and *Allorhizobium–Neorhizobium–Pararhizobium–Rhizobium*) only detected in plant-fed groups. Additionally, current findings suggest that gut microbial genera are more prevalent when *S. frugiperda* larvae feed on rice plants than on corn plants or an artificial diet and are closely linked to their metabolic activities. Dominant microbial genera are expected to support essential metabolic processes and exhibit increased abundance on rice. These results indicate that the gut microbiome of *S. frugiperda* is diet-driven reorganization, potentially facilitating its polyphagy. This study extends the current understanding by elucidating the specific gut microbial taxa and their putative metabolic associations that are responsive to diet in *S. frugiperda*, thereby providing a theoretical basis for its polyphagous capability and underscoring microbiota-based strategies for sustainable pest management.

## Simple Summary

*Spodoptera frugiperda* (J.E. Smith) is a highly polyphagous and globally invasive pest species that has inflicted significant damage on crop yields throughout China. Similar to other insects, *S. frugiperda* possesses numerous symbiotic bacteria that are crucial for host feeding, adaptability, and development. To elucidate the connections among host plants, insects, and microorganisms, we used high-throughput sequencing to examine the gut bacterial populations of *S. frugiperda* cultivated on several host plants. According to our research, the type of host plant has a significant impact on the diversity and composition of *S. frugiperda* gut bacteria. These findings offer essential baseline information for future research on gut microbial functions and their role in this pest’s extraordinary host adaptation capabilities.

## 1. Introduction

Fall armyworms (*Spodoptera frugiperda* J.E. Smith) are migratory, polyphagous pests native to the Americas. Owing to its broad host range, high reproductive capacity, and rapid dispersal, it has emerged as a global threat to the growth of agricultural crops [1,2]. Within the past decade, it has invaded large parts of Africa, Asia, and Oceania, infesting important staple crops such as sugarcane, rice, sorghum, and maize [3,4]. Its extensive larval feeding directly reduces yields, while its management has increased reliance on synthetic insecticides, thereby elevating costs and environmental risks and promoting insecticide resistance [5,6]. Comprehension knowledge of the biology and ecology of *S. frugiperda*, particularly its interactions with symbiotic bacteria, is critical to developing novel and sustainable pest management measures.

An important yet often overlooked aspect of insect biology is the gut microbiota, a diverse population of microorganisms residing in the digestive system that play a vital role in host growth, immunity, and plant adaptability [2,7]. Insects, particularly lepidopterans, harbor gut microbiota that contribute to plant digestion, xenobiotic detoxification, nutrition synthesis, and defense against pathogens [8,9]. The composition and function of these microbial communities are greatly influenced by a multitude of factors, including host genetics, developmental stage, environment, and diet [10]. Previous studies have demonstrated that changes in the gut microbiota induced by dietary variation can profoundly affect insect physiology and performance, influencing traits such as growth rate, fecundity, and susceptibility to both biotic and abiotic stressors [11,12,13]. In recent years, additional insect microbial communities have been identified in a variety of pest species after feeding on diverse host plants [14,15,16], including *S. frugiperda* [17,18].

One possible reason for the process of *S. frugiperda* is its flexible gut microbial population, which adjusts to dietary changes and may contribute to its ability to thrive on a wide range of host plants [1,19]. Understanding the composition and characteristics of these microbial communities under different feeding conditions could help identify potential targets for microbiome-based pest management strategies while also providing valuable insights into the insect’s resilience. However, research on the gut microbiota of *S. frugiperda* remains limited, particularly regarding how its microbial populations are influenced by natural host plants as opposed to artificial laboratory diets, despite the growing interest in insect–microbe interactions.

The main goal of previous studies has been to identify the core gut bacterial taxa in *S. frugiperda* and characterize the dynamic shifts of the microbiota across the host’s life cycle [20,21]. However, little is known about how the relative abundance and diversity of these and other microorganisms vary in response to different dietary components, particularly in ecologically significant scenarios involving corn and rice, the pest’s two main host plants in many regions of the world. Furthermore, limited research has explored the functional effects of these microbial changes, such as how modifications in microbial composition impact metabolic pathways related to detoxification, nutrition acquisition, and digestion [22,23,24]. We hypothesized that the composition and diversity of gut microbial communities would differ significantly among diets, with natural plant-based diets (corn and rice) supporting a more diverse and functionally specialized microbiome compared to the artificial diet.

In this study, we employed high-throughput 16S rRNA gene sequencing to characterize the gut microbiota of *S. frugiperda* larvae reared on three different diets: artificial feed, corn plants, and rice plants. The primary objectives were to (1) assess the microbial diversity and richness within the larval gut across different dietary treatments while also determining the compositional differences in microbial communities at the phylum, family, and genus levels and (2) predict the potential metabolic functions of these microbial communities using bioinformatics tools based on the Kyoto Encyclopedia of Genes and Genomes (KEGG) database. We hypothesized that specific microbial taxa would exhibit strong associations with particular metabolic pathways, potentially facilitating the host’s capacity to metabolize complex plant-derived substrates or tolerate secondary metabolites. By integrating microbial community profiling with functional predictions, this study aimed to provide a comprehensive understanding of *S. frugiperda* diet–microbiota interactions and their implications for host adaptation and pest management strategies.

## 2. Materials and Methods

### 2.1. Insect Rearing

The field population of *S. frugiperda* larvae was initially collected in 2019 from cornfields in Ping Hu County, Zhejiang Province (Latitude: 30.705° N, Longitude: 121.118° E). Then, the collected FAW strain was reared for over three years on 15-day-old corn and rice plants, while the control insects were maintained on an artificial diet described by Kasten [25] under controlled conditions (26 ± 1 °C, 60–70% relative humidity, and a 16 L: 8 D photoperiod).

### 2.2. Sample Collection and DNA Extraction

Newly hatched larvae were reared with corn (*Zea mays*), rice (*Oryza sativa* L.), and an artificial diet at the Zhejiang Academy of Agricultural Sciences (Zhejiang, China). Then, the 4th instar larvae were surface-sterilized with 70% ethanol for 1 min and subsequently rinsed three times with sterile water on a clean bench. Larvae from each dietary treatment were collected from three independent treatments: corn-fed, rice-fed, and control (fed an artificial diet), with each treatment containing 15–20 individuals. The midgut was dissected in sterile phosphate-buffered saline under a stereoscope. Microbial genomic DNA was extracted with the DNeasy Blood and Tissue Kit (QIAGEN, Hilden, Germany) according to the manufacturer’s instructions. The concentration of DNA was measured with a NanoDrop2000 spectrophotometer (Thermo Fisher Scientific, Waltham, MA, USA).

### 2.3. 16S rRNA Gene Sequencing

The V3-V4 region of the 16S rRNA gene was amplified using an ABI GeneAmp^®^ 9700 PCR thermocycler (ABI, Foster City, CA, USA) with universal primers 338F (5′-ACTCCTACGGGAGGCAGCAG-3′) and 806R (5′-GGACTACHVGGGTWTCTAAT-3′) [26]. Each polymerase chain reaction contained 4 μL of 5×TransStart^®^ Fastpfu buffer, 2 μL of 2.5 mM dNTPs, 0.8 μL of 5 μmol/L primer F, 0.8 μL of 5 μmol/L primer R, 0.4 μL of TransStart^®^ Fastpfu DNA Polymerase, 10 ng of DNA template, and double-distilled H_2_O to 20 μL. The PCR conditions were as follows: 95 °C for 3 min; 27 cycles of 95 °C for 30 s and 55 °C for 30 s and 72 °C for 45 s; 72 °C for 10 min. Since the PCR was used for targeted amplification of the 16S rRNA gene prior to sequencing for identification, the PCR products were separated on 2% agarose gel, purified using the AxyPrep DNA Gel Extraction Kit (Axygen Biosciences, Union City, CA, USA), and quantified with the QuantiFluorTM-ST kit (Promega, Madison, WI, USA) according to the manufacturer’s instructions. Equimolar amounts of the purified PCR amplicons were pooled and subjected to paired-end sequencing on the Illumina NovaSeq PE250 platform (Illumina, San Diego, CA, USA), following standard procedures provided by Majorbio Bio-Pharm Technology Co. Ltd. (Shanghai, China).

The bioinformatic pipeline was selected based on well-established, robust, and reproducible standards for 16S rRNA gene analysis, ensuring reliable and comparable results for microbial community profiling. Quality filtering of the raw FASTQ files generated on the Illumina platform was carried out using FASTP (v0.19.6) [27], and paired-end reads were assembled with FLASH (version 1.2.11) [28]. Clustering of the sequences into operational taxonomic units (OTUs) was performed in UPARSE (version 11) at a 97% sequence similarity cutoff [29], and chimeric sequences were identified and removed using UCHIME [30]. Taxonomy assignment of OTUs was performed using the RDP Classifier (version 2.13) [31] against the Silva 16S rRNA database using the Bayesian algorithm method. In accordance with standard practices for 16S rRNA gene analysis, results are reported and discussed primarily at the genus level to ensure robust and conservative interpretation of community data. Taxonomic information of varying levels was obtained from QIIME (version 1.9.1) [32]. Using the sequence number in the sample with the fewest sequences as the standard of homogenization to obtain the normalized output data for subsequent analyses. Representative reads were taxonomically classified by aligning them to the SILVA database (version 138) using the q2-feature-classifier with default settings.

### 2.4. Data Analysis

Bioinformatics analysis of the gut microbiota was performed on the Majorbio Cloud Platform (http://cloud.majorbio.com). Each treatment group has three replicates. Gut microbial α-diversity was assessed using Mothur (version 1.30.2), with the Ace and Chao indices employed to estimate species richness, and the Shannon and Simpson indices used to evaluate community diversity [33]. One-way ANOVA followed by the Tukey–Kramer Honest Significant Difference test was used to assess the statistical significance of differences in α-diversity indexes between diets. The higher Shannon index or lower Simpson index indicates a higher diversity of the gut microbiota. The differences in gut microbial composition between diets were evaluated using the permutation multivariate analysis of variance (PERMANOVA) (with 999 permutations) using the Vegan v2.5-3 package. The β-diversity was estimated by first calculating the Bray–Curtis dissimilarity of OUTs using QIIME (v 1.9.1) [32]. This distance matrix was then used as input for principal co-ordinate analysis (PCoA) to visualize the overall structural differences in microbial communities between diets. One-way ANOVA was performed to determine the statistical significance of the two coordinates’ difference in the relative abundance of respective microbial genera prevalent in the gut of *S. frugiperda*. The functional potential of the gut microbiota was predicted using the Kyoto Encyclopedia of Genes and Genomes (KEGG) databases through PICRUSt2 v2.2.0 [34]. Differences in the abundance of microbial groups associated with specific functions between dietary treatments were assessed using one-way ANOVA. Relationships between gut microbial composition and predicted functions were evaluated with Spearman’s correlation (rho). Visualization of functional differences and correlations was performed using STAMP plots and clustered heatmaps with significance indicators, generated through the Majorbio Cloud platform at http://cloud.majorbio.com/page/tools.html (accessed on 28 August 2024). Statistical analyses were performed with R statistical software version 3.3.1 [35].

## 3. Results

### 3.1. Gut Microbial Diversity in S. frugiperda

**Figure 1:**
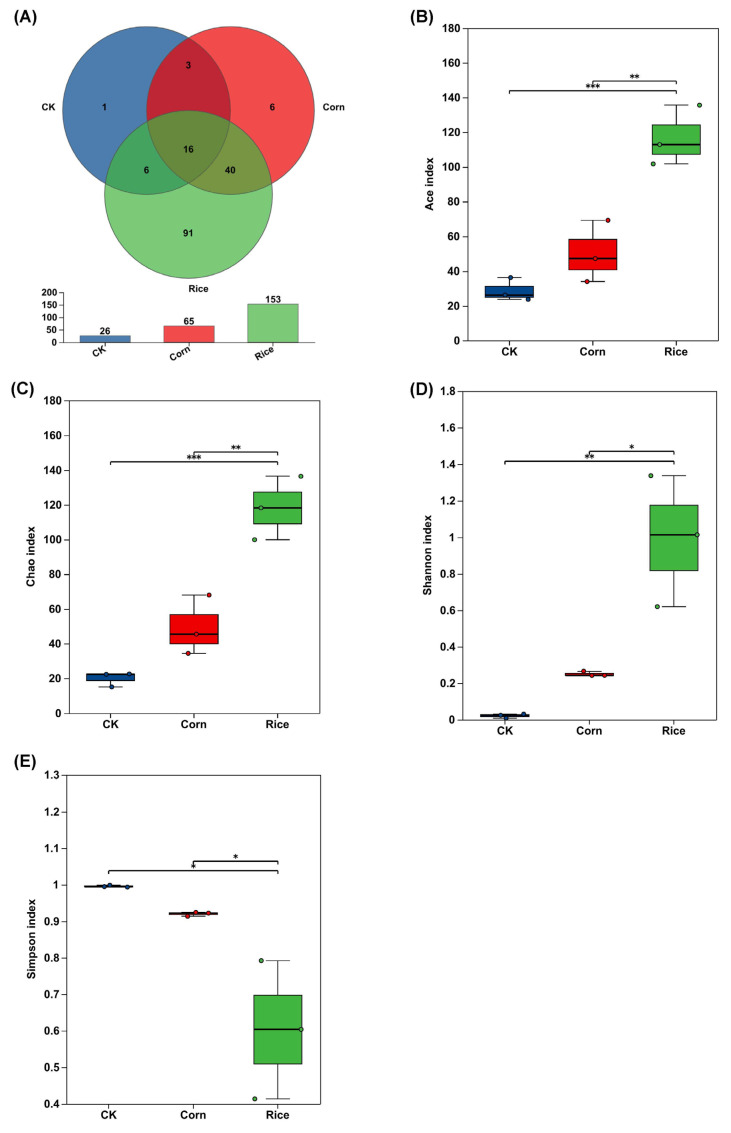
Microbial diversity in the gut regarding diet in *Spodoptera frugiperda*. (**A**) Numbers of microbial operational taxonomic units (OTUs). (**B**) Microbial diversity in ACE index. (**C**) Microbial diversity in the Chao index. (**D**) Microbial diversity in the Shannon index. (**E**) Microbial diversity in the Simpson index. Significant differences between diet groups were determined using one-way ANOVA followed by the Tukey–Kramer Honest Significant Difference test, with statistical significance denoted as follows: * *p* < 0.05, ** *p* < 0.01, and *** *p* < 0.001.

Across all samples from the corn population, rice population, and artificial diet-reared population, a total of 404,564 sequences were generated. After applying quality control measures and removing chimeric reads, an average of 44,952 high-quality sequences per sample was retained. At a 97% sequence similarity threshold, these sequences clustered into 163 operational taxonomic units (OTUs). Of these, six OTUs were unique to the corn-associated samples, ninety-one were exclusive to the rice-associated samples, and one was detected only in the control (Figure 1A, Table S1). We assess the community richness using the ACE and Chao1 indexes and the community diversity using the Shannon and Simpson indexes for the three groups. A maximum of 28 species were detected in the fall armyworm gut, and both the Shannon and Simpson indexes indicated that diversity was greatest in the gut of fall armyworms reared on the rice plants. Using a one-way ANOVA for comparison, we found that the richness and diversity indexes were significantly different (*p* < 0.05) between the artificial feed, corn, and rice samples (Figure 1B–E, Table S2).

### 3.2. Gut Microbial Composition in S. frugiperda

**Figure 2:**
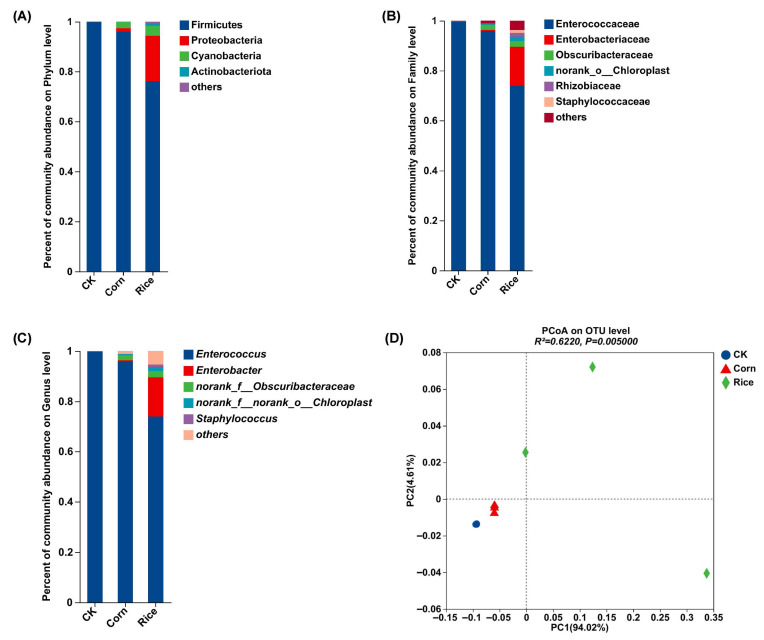
Co-occurrences of dominant gut microbes and their assemblages regarding diet in *Spodoptera frugiperda*. (**A**) Variation in taxonomic compositions of microbial communities in midgut collected from *Spodoptera frugiperda* reared on artificial feed, corn, and rice plants at the phylum level. (**B**) Taxonomic composition of microbial communities at the family level. (**C**) Taxonomic composition of microbial communities at the genus level. (**D**) Microbial assemblages of the three populations resulted from the principal co-ordinate analysis (PCoA) of Bray–Curtis dissimilarity among them.

Results showed that the dominant phyla included Firmicutes, Proteobacteria, Cyanobacteria, and Actinobacteriota, with Firmicutes accounting for over 99% in the control population. Specifically, the abundance of Firmicutes decreased from 95.98% in the corn population to 76.07% in the rice population, while the abundance of Proteobacteria increased from 1.44% in the corn population to 18.28% in the rice population (Figure 2A). At the family level, Enterococcaceae, Enterobacteriaceae, Obscuribacteraceae, norank_o_Chloroplast, and Rhizobiaceae were the top five families, among which Enterococcaceae take up the highest proportion in all three populations. The abundance of Enterobacteriaceae increased from 0.37% in the corn population to 15.57% in the rice population (Figure 2B). At the genus level, *Enterococcus* occupied almost the entire midgut of the control population, and *Enterococcus*, *Enterobacter*, *norank_f_Obscuribacteraceae*, *norank_f_norank_o_Chloroplast*, and *Staphylococcus* were the most abundant genera in the corn and rice populations. A higher abundance of *Enterobacter* in the rice population belongs to the family Enterobacteriaceae (Figure 2C). Analysis of microbial genus abundances revealed a significant difference between the three populations (Adonis, *p* = 0.005), with principal coordinates analysis (PCoA) showing that their microbial communities were clearly separated along the first principal component (Figure 2D).

**Figure 3:**
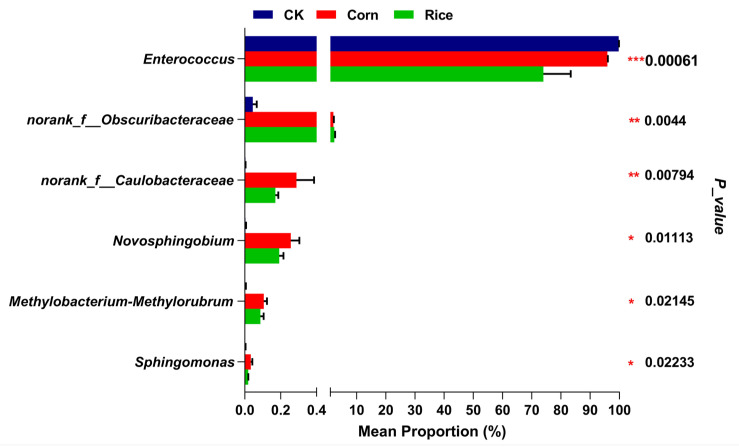
Variation in the relative abundance of dominant gut microbial genera in *Spodoptera frugiperda* was assessed with respect to diet. The x-axis is presented in two segments to clearly visualize both high- and low-abundance proportions. The left segment shows abundances from 0 to 0.4%, and the right segment shows an expanded view from 1% to 100%. Error bars indicate one standard deviation from the mean, and *p*-values shown in the right column were calculated using the one-way ANOVA, with statistical significance denoted as follows: * *p* < 0.05, ** *p* < 0.01, and *** *p* < 0.001.

Comparison among the most abundant genera revealed six genera that showed significant differences in their relative abundances across the gut microbiota of the three *S. frugiperda* populations. *Enterococcus* was the most abundant genus in the gut of the three populations; their relative abundances differed between the control and rice populations but were similar in the control and corn populations as well as the corn and rice populations. For some microbial groups such as *norank_f__Obscuribacteraceae*, *Novosphingobium*, and *Methylobacterium–Methylorubrum*, their relative abundances were significantly lower in the control population but were similar in the corn and rice populations. The relative abundances of some other prevalent groups, such as *norank_f__Caulobacteraceae* and *Sphingomonas,* were significantly more abundant in the corn population than in the control and rice populations (Figure 3, Table S3).

### 3.3. Functional Predictions of Gut Microbial Groups in S. frugiperda

**Figure 4:**
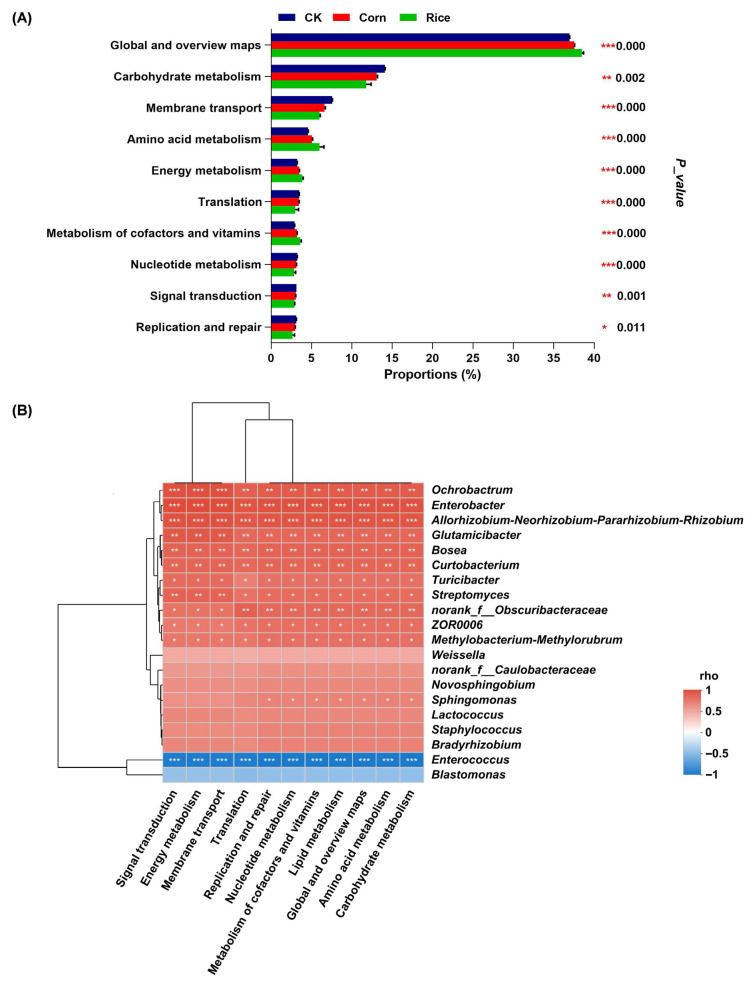
Predicted metabolic functions of gut microbiota in *Spodoptera frugiperda* on different diets. (**A**) Comparisons in relative abundance of predicted KEGG level 2 pathways across the three diets (one-way ANOVA). (**B**) Heatmap of Spearman’s correlation coefficients (rho) between the 20 most abundant microbial genera and the predicted abundance of significant metabolic functions. Correlation strength is indicated by color; the significance is denoted by asterisk (*, *p* < 0.05; **, *p* < 0.01; and ***, *p* < 0.001).

The majority of gut microbial groups were primarily involved in metabolic functions, while others were linked to environmental information processing, genetic information processing, human diseases, cellular processes, and organismal systems (Table S4). At the level 2 pathway analysis, six metabolic pathways were found to be more abundant in *S. frugiperda* fed an artificial diet compared to those reared on corn or rice plants. In contrast, four metabolic pathways showed higher prevalence in larvae fed on rice plants than on corn or the artificial diet (Figure 4A). Correlation analysis revealed that these pathways were positively associated with twelve dominant microbial genera, including *Ochrobactrum*, *Enterobacter*, *Allorhizobium–Neorhizobium–Pararhizobium–Rhizobium*, and *Glutamicibacter*; negatively associated with *Enterococcus*; and showed no significant correlation with seven genera, such as *Lactococcus*, *Staphylococcus*, and *Bradyrhizobium* (Figure 4B).

## 4. Discussion

The microbial ecosystem inhabiting insect digestive systems significantly influences host biological processes, encompassing critical functions such as nutrient breakdown, metabolic detoxification, immune response, and environmental adaptation. Focusing on the fall armyworm, *S. frugiperda*, our research utilized advanced metagenomic sequencing techniques to investigate how dietary variations impact gut microbial composition. Comprehensive molecular analysis revealed that nutritional sources—specifically corn, rice, and synthetic feed—substantially modulate the bacterial community structure within the fall armyworm’s gastrointestinal tract. Dietary components emerged as the primary determinant of microbial diversity, demonstrating remarkable plasticity in the insect’s gut microbiome. The composition of a limited number of highly abundant taxa aligns with the structure of gut bacterial communities observed in other insect species [12,13,36]. A previous study about Lepidoptera microbiota indicates that *Enterococcus* has been identified in approximately 70% of the species examined, while *Klebsiella* has been found in around 35% [37]. Our findings indicate that these two genera were also present in the core microbiota of *S. frugiperda*. Additionally, we observed a significant number of unclassified OTUs belonging to the family Enterobacteriaceae, which have been reported to be associated with over 80% of Lepidoptera species studied to date [37]. It is important to emphasize that while we identified OTUs as *Enterococcus* with bootstrap values close to 100%, the OTUs identified as *Klebsiella* likely represent a different genus due to their relatively low bootstrap value of 67%. This uncertainty regarding *Klebsiella*, along with the presence of ubiquitous but unclassified Enterobacteriaceae, highlights the need for more thorough characterization of microorganisms associated with Lepidoptera. Such efforts will enable us to make more meaningful comparisons between these microorganisms and their environmental counterparts as well as across various host species.

Additionally, we observed substantial differences in gut microbial community structures among different hosts. Notably, *S. frugiperda* reared on rice displayed significantly higher gut microbial richness and diversity, as evidenced by the Ace, Chao, and Shannon indices, along with a lower Simpson index. This trend is consistent with prior research on other Lepidoptera, such as *Spodoptera exigua*, *Antheraea pernyi*, and *Antheraea yamamai*, where shifts in host plants significantly affected microbial diversity [12,15]. Interestingly, rice-fed larvae displayed unique OTUs compared to corn-fed larvae, indicating that rice has a more substantial influence on gut microbiota composition. Notably, the PCoA revealed greater within-group variation in the rice-fed larvae compared to the corn and artificial diet groups. This pattern may reflect the higher heterogeneity of the rice diet, increased physiological variation among larvae on a potentially suboptimal host, and/or a greater influence of stochastic assembly processes in shaping the gut microbiota under these dietary conditions. At the phylum level, Firmicutes, Actinobacteriota, and Proteobacteria were dominant across all treatments, which aligns with findings from earlier studies on *S. frugiperda*, *Spodoptera litura*, and *P. xylostella* [19,38,39]. Firmicutes remained the dominant phylum across all groups but exhibited a decrease in relative abundance from corn-fed larvae (95.98%) to rice-fed larvae (76.07%). In contrast, Proteobacteria displayed an opposite trend, increasing in abundance among corn-fed larvae compared to those fed rice. This shift highlights the plasticity and adaptability of the microbiota in response to varying host-derived diets.

Firmicutes play a crucial role in nutrient metabolism, particularly due to their capacity to degrade complex carbohydrates and synthesize sugar transporters, which might help mitigate the effects of toxic secondary plant metabolites [8,39,40]. The consistently high abundance of Firmicutes underscores their essential role in the digestion and metabolism of *S. frugiperda*. Additionally, Actinobacteriota were notably more abundant in larvae fed rice and wheat, consistent with previous research linking these bacteria to extracellular enzyme production and antimicrobial activity [17]. At the family level, Enterococcaceae was the predominant group across all populations, especially in the control group. This dominance reinforces their central role in the gut microbiomes of Lepidoptera, highlighting their importance in the overall microbial community structure within these insects [2,41]. Enterococcaceae are associated with plant cell wall degradation and detoxification of plant allelochemicals [42,43].

Importantly, Enterobacteriaceae, especially *Enterobacter* and *Klebsiella*, were more prevalent in rice-fed and corn-fed larvae. These genera have known roles in cellulose degradation and glucose metabolism [44,45] and may compensate for the challenges posed by cellulose-rich or chemically defensive diets. *Klebsiella*, in particular, is a beneficial symbiont known to enhance insect development and fecundity by suppressing plant defenses and enhancing nutrient absorption [43,46]. Their high abundance in rice-, wheat-, and honeysuckle-fed larvae corresponds with previous observations of shorter development time and higher survival rates in these groups [17], suggesting a tight host–microbe–diet interaction that facilitates adaptation and reproductive fitness. Therefore, targeted manipulation of beneficial microbes and integration into integrated pest management programs (IPM) may be a future research direction. Although we did not evaluate insect-related characteristics (such as body weight) or conduct non-target metabolomic comparisons among diets, our research indicates that dietary variations may have an impact on insect growth and metabolic processes. We will include these results in future research in order to provide a deeper understanding of how nutrition affects insect physiology.

Interestingly, dietary variation also significantly influenced the prevalence of less-studied taxa within the larval gut. For example, *Curtobacterium*, *Staphylococcus*, and *Ochrobactrum* exhibited substantially higher abundance in rice-fed larvae than in other diet groups. Functional prediction analyses revealed positive correlations between genera, such as *Ochrobactrum*, *Allorhizobium*, *Neorhizobium*, *Pararhizobium*, *Rhizobium*, and *Glutamicibacter,* and several key metabolic pathways. These pathways, predominantly involved in diverse metabolic processes, displayed heightened activity in rice-fed larvae and showed negative correlations with *Enterococcus* dominance, suggesting potential functional trade-offs within the microbial community based on dietary input. It is important to note that the functional predictions presented here, derived from 16S rRNA gene data using PICRUSt2, are inferential in nature. While this approach provides valuable hypotheses regarding potential metabolic capabilities, these predictions are based on phylogenetic inference and reference genomes and do not directly measure microbial gene expression or metabolic activity. Future studies incorporating metatranscriptomic, metaproteomic, or biochemical assays are necessary to experimentally validate these proposed functional roles. Furthermore, Principal Coordinate Analysis (PCoA) and Adonis testing provided further statistical support, confirming significant separation of microbial assemblages among the rice, corn, and control groups (*p* = 0.005). This reinforces the conclusion that diet is a primary driver of gut microbial community structure in *S. frugiperda*. These distinctions at both the taxonomic and functional levels reveal a complex yet consistent pattern of diet-driven microbial reshaping, which likely influences insect development, metabolic processes, and adaptability to different host plants.

Based on our current findings, future research should focus on functional validation of key bacterial taxa and host genes involved in gut–microbe interactions, using approaches such as RNA interference (RNAi), CRISPR-based gene editing, or targeted knockdown strategies. Experimental manipulation of the gut microbiota (for example, through antibiotics, probiotics, etc.) could further clarify the links between dietary shifts and microbial community dynamics. Such integrative approaches would provide mechanistic insights into how diet-mediated microbial changes influence the adaptability of *S. frugiperda* and could inform the development of novel, microbial-based pest management strategies.

## 5. Conclusions

Taken together, our findings demonstrate that host plant diet exerts a profound influence on the gut microbiota of *S. frugiperda*, revealing clear shifts in microbial diversity and composition associated with different feeding regimes. These results provide new insights into how plant–insect–microbe interactions shape host adaptability and may underlie the pests’ remarkable polyphagy. By identifying key bacterial taxa potentially linked to host plant preference and metabolic adaptation, this study highlights promising avenues for microbiota-based pest management strategies aimed at disrupting pest survival on specific crops in a sustainable and environmentally friendly manner. Current findings lay a foundation for developing long-term, ecologically sound pest control strategies. Future research should extend these microbiome findings using functional approaches such as RNAi, CRISPR-based gene editing, or targeted manipulation of the gut microbiota to validate the roles of key bacterial taxa and host genes in *S. frugiperda*’s dietary adaptation and to inform innovative control methods.

## Data Availability

The original contributions presented in this study are included in the article/Supplementary Material. Further inquiries can be directed to the corresponding authors.

## Supplementary Materials

The following supporting information can be downloaded at: https://www.mdpi.com/article/10.3390/insects16101042/s1, Table S1: EXCEL sheet of gut microbial compositions in *S. frugiperda*; Table S2: Contrasts of gut microbial richness and diversity indexes across three diets in *S. frugiperda*; Table S3: *p*-values (one-way ANOVA) from contrasts in abundance across three diets for six prevalent gut microbial genera in *S. frugiperda*; Table S4: EXCEL spreadsheet of KEGG function predictions in pathway at level 1.
