# Supplementary material for: Influence of Host’s Plant Diet on Gut Microbial Communities and Metabolic Potential in Spodoptera frugiperda
Source: Insects. 2025 Oct 10;16(10):1042. doi: 10.3390/insects16101042 (PMC12565223; doi:10.3390/insects16101042)
Supplement: Supplementary file 1 [file insects-16-01042-s001.zip › Table S2.pdf]

Table S2. Contrasts of gut microbial richness and diversity indexes across three diets in *S. frugiperda.*

ID	Threshold	Coverage	Number of OTUs	Alpha diversity			
				ACE	Chao	Shannon	Simpson
CK_1	0.03	0.999878	7	36.32	15.00	0.0079	0.9984
CK_2	0.03	0.999878	14	23.71	22.50	0.0302	0.9932
CK_3	0.03	0.999829	10	26.22	22.25	0.0238	0.9945
Corn_1	0.03	0.999586	20	69.20	68.00	0.2425	0.9218
Corn_2	0.03	0.999878	19	34.02	34.33	0.2655	0.9134
Corn_3	0.03	0.999756	22	47.13	45.43	0.2423	0.9239
Rice_1	0.03	0.999561	28	101.72	99.91	1.3369	0.4135
Rice_2	0.03	0.999391	27	112.88	118.27	0.6193	0.7919
Rice_3	0.03	0.999634	27	135.64	136.50	1.0139	0.6033
P value			<0.001	0.0085	0.0085	0.0002	0.0005

OTUs were defined at the 97% similarity level (the threshold is 0.03).

The abbreviation CK, Corn, and Rice stands for *S. frugiperda* reared on artificial feed, corn and rice plants, respectively.

$P < 0.05$ is considered a significant difference.
