# Supplementary material for: Influence of Host’s Plant Diet on Gut Microbial Communities and Metabolic Potential in Spodoptera frugiperda
Source: Insects. 2025 Oct 10;16(10):1042. doi: 10.3390/insects16101042 (PMC12565223; doi:10.3390/insects16101042)
Supplement: Supplementary file 1 [file insects-16-01042-s001.zip › Table S3.pdf]

Table S3. *P*-values (One-way ANOVA) from contrasts in abundance across three diets for six prevalent gut microbial genera in *S. frugiperda.*

Genus	CK-Corn	CK-Rice	Corn-Rice
Enterococcus	≥ 0.1	< 0.05	< 0.1
norank_f__Obscuribacteraceae	< 0.001	< 0.001	≥ 0.1
norank_f__Caulobacteraceae	< 0.05	≥ 0.1	≥ 0.1
Novosphingobium	< 0.01	< 0.05	≥ 0.1
Methylobacterium-Methylorubrum	< 0.01	< 0.05	≥ 0.1
Sphingomonas	< 0.05	≥ 0.1	≥ 0.1

The abbreviation CK, Corn, and Rice stands for *S. frugiperda* reared on artificial feed, corn and rice plants, respectively.
